# Correction: Rheotaxis facilitates upstream navigation of mammalian sperm cells

**DOI:** 10.7554/eLife.03521

**Published:** 2014-06-03

**Authors:** Vasily Kantsler, Jörn Dunkel, Martyn Blayney, Raymond E Goldstein

Kantsler V, Dunkel J, Blayney M, Goldstein RE. 2014. Rheotaxis facilitates upstream navigation of mammalian sperm cells. *eLife* **3**:e02403. doi: http://dx.doi.org/10.7554/eLife.02403. Published 27 May 2014

In the published article, the Excel document intended as Supplementary file 2 was accidentally uploaded as Supplementary file 1, and the PDF intended as Supplementary file 1 was omitted.

The files and relevant citations in the text have now been corrected.

