# Supplementary material for: Rheotaxis facilitates upstream navigation of mammalian sperm cells
Source: eLife. 2014 May 27;3:e02403. doi: 10.7554/eLife.02403 (PMC4031982; doi:10.7554/eLife.02403)
Supplement: Supplementary file 1. — This file contains a detailed mathematical derivation of the minimal model in Equations 1 and 2 of the main text, a description of the parameter estimation procedure and a brief summary of numerical methods. DOI: http://dx.doi.org/10.7554/eLife.02403.013 [file elife02403s001.pdf]

# Supplementary File 1 for 'Rheotaxis facilitates upstream navigation of mammalian sperm cells': Mathematical modeling and parameter estimation

Vasily Kantsler,[1] Jörn Dunkel,[2] Martyn Blayney,[3] and Raymond E. Goldstein[4,*]

*[1]University of Warwick, Gibbet Hill Road, Coventry, CV4 7AL, UK*
*[2]Department of Mathematics, Massachusetts Institute of Technology, 77 Massachusetts Avenue E17-412, Cambridge, MA 02139-4307, USA*
*[3]Bourn Hall Clinic, Bourn, Cambridge CB23 2TN, UK*
*[4]Department of Applied Mathematics and Theoretical Physics, University of Cambridge, Wilberforce Road, Cambridge CB3 0WA, UK*

## I. THEORY

### A. Structure of the 2D equations of motion

We would like to deduce the general structure of the simplified 2D equations of motion governing the reorientation sperm swimming under shear flow close to a solid surface. It is useful to consider non-chiral particles first. Corrections due to chirality will be discussed subsequently.

#### 1. Non-chiral objects

We assume a geometry as depicted in Fig. 1B of the Main Text and shear-flow near the boundary given by

$$\boldsymbol{u} = \begin{pmatrix} 0 \\ \sigma\dot{\gamma}z \\ 0 \end{pmatrix}, \tag{1}$$

where $\dot{\gamma} > 0$ is the shear rate and $\sigma = \pm 1$ depending on flow direction. The corresponding vorticity pseudo-vector $\boldsymbol{\omega}$ and rate-of-strain tensor $\mathcal{E}$ read

$$\boldsymbol{\omega} = \nabla \times \boldsymbol{u} = -\sigma\dot{\gamma} \begin{pmatrix} 1 \\ 0 \\ 0 \end{pmatrix}, \qquad \mathcal{E} = \frac{1}{2}\left(\nabla^{\mathsf{T}}\boldsymbol{u} + \nabla\boldsymbol{u}^{\mathsf{T}}\right) = \frac{\sigma\dot{\gamma}}{2} \begin{pmatrix} 0 & 0 & 0 \\ 0 & 0 & 1 \\ 0 & 1 & 0 \end{pmatrix}. \tag{2}$$

We describe the orientation of the cells by the 3D orientation vector $\boldsymbol{n} = (n_x, n_y, n_z)$ and denote the associated orthogonal projector by

$$\mathcal{P}(\boldsymbol{n}) = \mathcal{I} - \boldsymbol{n}\boldsymbol{n}, \tag{3}$$

where $\mathcal{I}$ is the $3 \times 3$ unit matrix. According to Eq. (2.4) of Pedley & Kessler [1], reorientation of elliptical (or rod-like) particles in shear flow $\boldsymbol{u}$ is governed to leading order by

$$\dot{\boldsymbol{n}} = a\boldsymbol{\omega} \times \boldsymbol{n} + 2b\boldsymbol{n} \cdot \mathcal{E} \cdot \mathcal{P}(\boldsymbol{n}), \tag{4}$$

where $a = 1/2$ and $b = 0$ for spherical particles. Note that the structure of Eq. (6) is such that it conserves the length of $\boldsymbol{n}$.

Moreover, we may identify the cross-product $\boldsymbol{\omega} \times \boldsymbol{n}$ with a matrix multiplication $\mathcal{W} \cdot \boldsymbol{n}$, where the components of the antisymmetric matrix $\mathcal{W}$ are given by

$$\begin{aligned} 2\mathcal{W}_{mn} &:= -(\boldsymbol{\omega}\times)_{mn} \\ &= -\epsilon_{min}(\epsilon_{ijk}\partial_j u_k) = \epsilon_{imn}(\epsilon_{ijk}\partial_j u_k) = (\delta_{mj}\delta_{nk} - \delta_{mk}\delta_{nj})\partial_j u_k \\ &= \partial_m u_n - \partial_n u_m, \end{aligned} \tag{5}$$

*Electronic address: R.E.Goldstein@damtp.cam.ac.uk

using a component notation with sum convention for repeated Latin indices $i, j, \ldots = 1, 2, 3$. This allows us to rewrite Eq. (4) as

$$\dot{n}_i = 2a\mathcal{W}_{ij} n_j + 2b n_m \mathcal{E}_{mj}(\delta_{ji} - n_j n_i). \tag{6}$$

For the flow field in (1) we find

$$\dot{\boldsymbol{n}} \;=\; -a\sigma\dot{\gamma}\begin{pmatrix} 0 \\ -n_z \\ n_y \end{pmatrix} - b\sigma\dot{\gamma}\begin{pmatrix} 2n_x n_y n_z \\ (2n_y^2 - 1)n_z \\ (2n_z^2 - 1)n_y \end{pmatrix}, \tag{7}$$

with $a, b$ encoding information about the cell-shape.

Now assume that after a collision with the boundary and subsequent alignment, the sperm points into the wall due to steric contact interactions between surface and flagellum, so that $n_z = \mathit{const} < 0$. This means that the wall must exert a balancing torque such that (i) $\dot{n}_z = 0$ and (ii) $n_x^2 + n_y^2 = (1 - n_z^2)$ is conserved. Assuming cylindrical symmetry of the swimmer around its axis of swimming, the contact interaction leads to rotation of the swimmer in the plane spanned by $\boldsymbol{n}$ and the wall normal $\hat{\boldsymbol{z}} = (0, 0, 1)$. The change in orientation per unit time due to wall interactions can therefore be written as $c\boldsymbol{n} + d\hat{\boldsymbol{z}}$, which needs to be added to the rhs. of Eqs. (4), (6) and (7), if the sperm is contact with the surface. The coefficient $d$ can be fixed by condition (i), but is not relevant for the motion in the $(x, y)$-plane parallel to the surface, which becomes governed by

$$\begin{pmatrix} \dot{n}_x \\ \dot{n}_y \end{pmatrix} = -a\sigma\dot{\gamma}n_z\begin{pmatrix} 0 \\ -1 \end{pmatrix} - b\sigma\dot{\gamma}n_z\begin{pmatrix} 2n_x n_y \\ 2n_y^2 - 1 \end{pmatrix} + c\begin{pmatrix} n_x \\ n_y \end{pmatrix}. \tag{8}$$

Condition (ii) then gives

$$c = \sigma\dot{\gamma}\frac{n_z[b(1 - 2n_z^2) - a]}{1 - n_z^2} n_y. \tag{9}$$

Keeping in mind that $n_z$ and $n_x^2 + n_y^2$ are constant, we thus find the reduced 2D equations of motion

$$\begin{pmatrix} \dot{n}_x \\ \dot{n}_y \end{pmatrix} = -\sigma\dot{\gamma}(a + b)\frac{n_z}{n_x^2 + n_y^2}\begin{pmatrix} n_x n_y \\ n_y^2 - (1 - n_z^2). \end{pmatrix} \tag{10}$$

The fixed point criterium $(\dot{n}_x, \dot{n}_y) = 0$ gives

$$n_x = 0, \qquad\qquad n_y = \pm\sqrt{1 - n_z^2}, \tag{11}$$

This result implies that, depending on the effective shape parameter

$$\alpha = -(a + b)n_z, \tag{12}$$

the combination of shear flow and wall interaction aligns the swimmer either parallel or anti-parallel to the flow direction. This result also indicates that, in order to account for the transverse velocity component observed in the experiments, we also need to consider the chirality of the flagellar beat, which has been neglected so far. Before discussing chiral effects in the next section, let us still note that we may rewrite Eq. (10) in terms of the 2D unit vector

$$\boldsymbol{N} = \begin{pmatrix} N_x \\ N_y \end{pmatrix} = \frac{1}{\sqrt{1 - n_z^2}}\begin{pmatrix} n_x \\ n_y \end{pmatrix} \tag{13}$$

as

$$\begin{pmatrix} \dot{N}_x \\ \dot{N}_y \end{pmatrix} = \sigma\dot{\gamma}\alpha\begin{pmatrix} N_x N_y \\ N_y^2 - 1 \end{pmatrix}, \tag{14}$$

where $\sigma = \pm 1$ accounts for the flow direction and constant geometric prefactors have been absorbed in the 'shape' coefficient

$$\alpha = -(a + b)n_z. \tag{15}$$

Note that $\alpha$ is positive for sperm-type swimmers that point into the surface, for in this case one has $n_z < 0$.

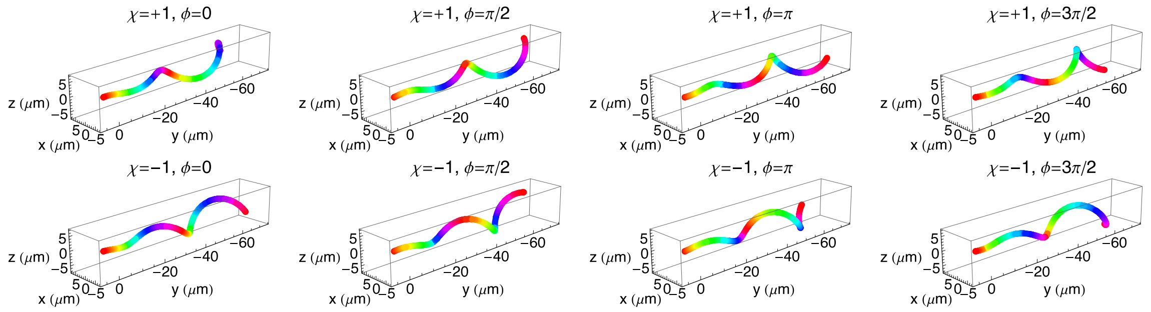


FIG. 1: Rigid conical helices, as defined in Eq. (16), for different handedness $\chi = \pm 1$ and different 'initial' phases $\phi$ in their body-fixed frames, where the head rests at the origin. Colors encode windings. Parameters: $S = 4\pi$, $\epsilon_1 = \epsilon_2 = 0.1$, $\lambda = 5\mu$m.

### 2. *Chiral objects*

To identify how chirality of the flagellar beat might affect the reorientation rate of sperm in shear flow, we consider as a simplified sperm model a rigid 3D conical helix $\boldsymbol{C}(s)$ in contact with a wall that defines the $(x, y)$-plane of the lab frame $\Sigma = \{\boldsymbol{e}_x, \boldsymbol{e}_y, \boldsymbol{e}_z\}$, which is again chosen as in Fig. 1B of the Main Text.

We denote by $\boldsymbol{n} = (n_x, n_y, n_z)$ the orientation of the conical helix in the lab frame $\Sigma$. The head position is identified with tip of the cone. In the body-fixed frame $\hat{\Sigma} = \{\hat{\boldsymbol{e}}_x, \hat{\boldsymbol{e}}_y, \hat{\boldsymbol{e}}_z\}$, the head rests at the origin and the tail points in the $-\hat{\boldsymbol{e}}_y$-direction (Fig. **??**). Specifically, we assume that in the body-fixed frame $\hat{\Sigma}$ the helix is described by the curve

$$\hat{\boldsymbol{C}}(s) = \begin{pmatrix} \hat{X}(s) \\ \hat{Y}(s) \\ \hat{Z}(s) \end{pmatrix} = \lambda s \begin{pmatrix} \chi\epsilon_1 \cos(s-\phi) \\ -1 \\ -\epsilon_2 \sin(s-\phi) \end{pmatrix}, \qquad s \in [0, S]. \tag{16}$$

The length parameter $\lambda$ scales the size of the flagellum. The parameters $\epsilon_1 > 0$ and $\epsilon_2 > 0$ determine the lateral shape of the helix, and they can also be used to interpolate between helical and planar beat patterns. The phase angle $\phi$ sets the 'initial' direction of the flagellum relative to the head. The chirality parameter $\chi = \pm 1$ determines the handedness, defined here such that $\chi = +1$ corresponds to a right-handed spiral when viewed from the head (Fig. 2).

To simplify calculations, we henceforth focus on symmetric spirals with $\epsilon_1 = \epsilon_2 = \epsilon$. In this case, the enveloping cone is given by

$$\hat{\mathfrak{C}}(s, \phi) = \lambda s \begin{pmatrix} \epsilon \cos\phi \\ -1 \\ -\epsilon \sin\phi \end{pmatrix}, \qquad s \in [0, S],\ \phi \in [0, 2\pi) \tag{17}$$

with half-opening angle

$$\theta_\epsilon = \arctan \epsilon. \tag{18}$$

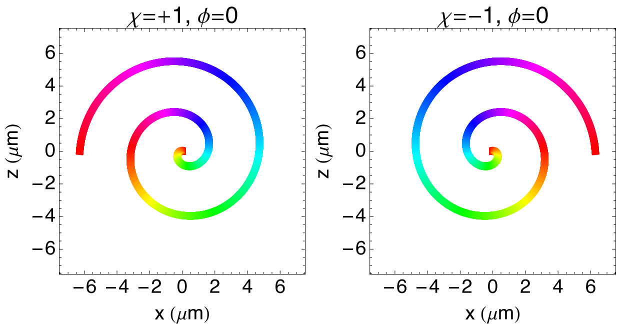


FIG. 2: Rigid conical helices from Fig. 1 as viewed from the head, using the same color coding as in Fig. 1.

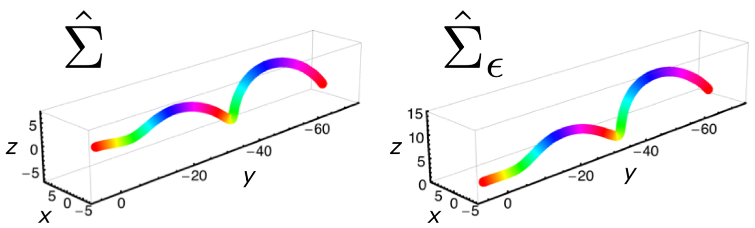


FIG. 3: Body-centered frame $\hat{\Sigma}$ and tilted body-centered frame $\hat{\Sigma}_\epsilon$ are related by a rotation about the $x$-axis. After alignment with the wall, which is assumed to lie in the $(z=0)$-plane of $\hat{\Sigma}_\epsilon$, the orientation of the sperm in the lab frame $\Sigma$ (not shown) is obtained by an additional rotation of $\hat{\Sigma}_\epsilon$ about the vertical axis .

Assume that the spiral is in contact with the surface along its envelope. By rotating through $-\theta$ about the $\hat{\boldsymbol{e}}_x$-axis, we obtain the tilted body-centered frame $\hat{\Sigma}_\epsilon$ (Fig. 3) which is defined such that the channel surface is located at $z=0$ in both $\hat{\Sigma}_\epsilon$ and the lab frame. In $\hat{\Sigma}_\epsilon$, the helix is given by

$$\hat{\boldsymbol{C}}_\epsilon(s) = \mathcal{R}_x(\theta_\epsilon)\cdot\hat{\boldsymbol{C}}(s), \qquad \mathcal{R}_x(\theta_\epsilon) = \begin{pmatrix} 1 & 0 & 0 \\ 0 & \cos\theta_\epsilon & \sin\theta_\epsilon \\ 0 & -\sin\theta_\epsilon & \cos\theta_\epsilon \end{pmatrix}. \tag{19}$$

Using $\chi^2 = 1$, the tangent vectors in the body-fixed frame $\hat{\Sigma}$ are found as

$$\begin{aligned} \hat{\boldsymbol{t}}(s) &:= \frac{d\hat{\boldsymbol{C}}(s)/ds}{||d\hat{\boldsymbol{C}}(s)/ds||} \\ &= \frac{1}{\sqrt{1+(1+s^2)\,\epsilon^2}} \begin{pmatrix} \epsilon\chi[\cos(s-\phi) - s\sin(s-\phi)] \\ -1 \\ -\epsilon[s\cos(s-\phi)+\sin(s-\phi)] \end{pmatrix}. \end{aligned} \tag{20}$$

and, accordingly, after alignment with the wall in $\hat{\Sigma}_\epsilon$ as

$$\hat{\boldsymbol{t}}_\epsilon(s) = \mathcal{R}_x(\theta_\epsilon)\cdot\hat{\boldsymbol{t}}(s). \tag{21}$$

Due to our chosen parameterisation (16), the tangent vectors point away from the head. The length $\Lambda$ of the curve $\boldsymbol{C}(s)$ is obtained as

$$\begin{aligned} \Lambda &= \int_0^S ds \left|\left|\frac{d\hat{\boldsymbol{C}}(s)}{ds}\right|\right| \\ &= \int_0^S ds \sqrt{\hat{X}'(s)^2 + \hat{Y}'(s)^2 + \hat{Z}'(s)^2} \\ &= S\lambda\left[1+\frac{\epsilon^2}{6}(3+S^2)\right] + \mathcal{O}(\epsilon^4). \end{aligned} \tag{22}$$

Thus, to leading order, one can identify $\Lambda \simeq S\lambda$ with the length of a flagellum, and $A = \Lambda\epsilon$ with the beat amplitude.

After averaging over all initial conditions $\phi$, the mean geometric center of the helix in the body-fixed frame $\hat{\Sigma}_\epsilon$ is found as

$$\begin{aligned} \bar{\hat{\boldsymbol{C}}}_\epsilon &:= \frac{1}{2\pi}\int_0^{2\pi} d\phi \left[\frac{1}{\Lambda}\int_0^S ds \left|\left|\frac{d\hat{\boldsymbol{C}}(s)}{ds}\right|\right| \hat{\boldsymbol{C}}_\epsilon(s)\right] \\ &= \frac{S\lambda}{2}\begin{pmatrix} 0 \\ -1 \\ \epsilon \end{pmatrix} + \mathcal{O}(\epsilon^2). \end{aligned} \tag{23}$$

The orientation $\hat{\boldsymbol{n}}_\epsilon$ in the wall-aligned body-fixed frame $\hat{\Sigma}_\epsilon$ is defined by

$$\hat{\boldsymbol{n}}_\epsilon := -\frac{\bar{\hat{\boldsymbol{C}}}_\epsilon}{||\bar{\hat{\boldsymbol{C}}}_\epsilon||} = \begin{pmatrix} 0 \\ 1 \\ -\epsilon \end{pmatrix} + \mathcal{O}(\epsilon^2), \tag{24}$$

which is normalised up to terms of order $\mathcal{O}(\epsilon^2)$. Recalling that the $z$-axes of $\hat{\Sigma}_\epsilon$ and lab-frame $\Sigma$ coincide, the negative $z$-component means that the swimming direction points into the wall.

Let us assume, as before, that the shear fluid flow in the lab frame $\Sigma$ is along the $\boldsymbol{e}_y$-direction,

$$\boldsymbol{u} = \sigma\dot{\gamma}z\boldsymbol{e}_y, \tag{25}$$

where $\dot{\gamma} > 0$ is the shear rate and $\sigma = \pm 1$ determines the flow direction. Measuring the orientation angle $\psi$ of the swimmer wrt. $\boldsymbol{e}_y$ in counterclockwise direction, we obtain the coordinates $\boldsymbol{C}(t,s)$ of the helix with head position $\boldsymbol{R}(t) = (X(t), Y(t), 0)$ in the lab frame $\Sigma$ by

$$\boldsymbol{C}(t,s) = \boldsymbol{R}(t) + \mathcal{R}(\psi(t)) \cdot \hat{\boldsymbol{C}}_\epsilon(s), \tag{26}$$

where

$$\mathcal{R}(\psi) = \begin{pmatrix} \cos\psi & -\sin\psi & 0 \\ \sin\psi & \cos\psi & 0 \\ 0 & 0 & 1 \end{pmatrix} \tag{27}$$

represents a rotation about the $\boldsymbol{e}_z$-axis. By applying the rotation matrix $\mathcal{R}(\psi)$ to the orientation vector $\hat{\boldsymbol{n}}_\epsilon$ in $\hat{\Sigma}_\epsilon$, we find that, to leading order in $\epsilon$, the 3D orientation vector $\boldsymbol{n}$ in the lab frame $\Sigma$ is given by

$$\boldsymbol{n} = \begin{pmatrix} \boldsymbol{N} \\ -\epsilon \end{pmatrix} + \mathcal{O}(\epsilon^2), \qquad \boldsymbol{N} = \begin{pmatrix} N_x \\ N_y \end{pmatrix} = \begin{pmatrix} -\sin\psi \\ \cos\psi \end{pmatrix}, \tag{28}$$

where $\boldsymbol{N}$ is the normalised (projected) 2D orientation vector in the $(x, y)$-plane. This allows us to rewrite the rotation matrix as

$$\mathcal{R}_{\boldsymbol{N}} = \begin{pmatrix} N_y & N_x & 0 \\ -N_x & N_y & 0 \\ 0 & 0 & 1 \end{pmatrix}. \tag{29}$$

The tangent vectors of $\boldsymbol{C}$ in $\Sigma$ are given by $\boldsymbol{t}(s) = \mathcal{R}_{\boldsymbol{N}} \cdot \mathcal{R}_x(\theta_\epsilon) \cdot \hat{\boldsymbol{t}}(s)$ with $\hat{\boldsymbol{t}}(s)$ from Eq. (20).

Assuming that the head position $\boldsymbol{R}(t)$ of the helix performs a quasi-2D motion along the surface, $\boldsymbol{R}(t) = X(t)\boldsymbol{e}_x + Y(t)\boldsymbol{e}_y$, we are interested in obtaining simplified effective equations for the mean drag velocity $\dot{\boldsymbol{R}} = \boldsymbol{U}(\boldsymbol{N})$ and the change in the orientation $\dot{\boldsymbol{N}}(t)$ due to the action of the flow gradient on the rigid helical curve $\boldsymbol{C}$. As we shall discuss next, such equations can be derived from resistive force theory (RFT).

From Eq. (26), the velocity of some point $s \in [0, S]$ on the helix can be decomposed as[1]

$$\dot{\boldsymbol{C}}(s) = \dot{\boldsymbol{R}} + \dot{\mathcal{R}}_{\boldsymbol{N}} \cdot \hat{\boldsymbol{C}}_\epsilon = \boldsymbol{U} + \dot{\mathcal{R}}_{\boldsymbol{N}} \cdot \hat{\boldsymbol{C}}_\epsilon. \tag{30}$$

Given the shear flow profile $\boldsymbol{u}$, RFT assumes that the force line-density (force per unit length) can be split as

$$\begin{aligned} \boldsymbol{f}(s) \;=\; & \zeta_{||} \left\{ \left[ \boldsymbol{u}(\boldsymbol{C}(s)) - \dot{\boldsymbol{C}}(s) \right] \cdot \boldsymbol{t}(s) \right\} \boldsymbol{t}(s) + \\ & \zeta_\perp \left\{ \left[ \boldsymbol{u}(\boldsymbol{C}(s)) - \dot{\boldsymbol{C}}(s) \right] \cdot [\boldsymbol{I} - \boldsymbol{t}(s)\boldsymbol{t}(s)] \right\} \end{aligned} \tag{31}$$

where $\zeta_{||}$ and $\zeta_\perp$ are tangential and perpendicular drag coefficients. The drag ratio

$$\kappa = \frac{\zeta_\perp}{\zeta_{||}}, \tag{32}$$

which equals 2 for rigid rods, takes values $\kappa \simeq 1.4 - 1.7$ for realistic flagella. Combining the RFT ansatz (31) with the zero-force and zero-torque conditions of the over-damped Stokes-regime

$$0 \;=\; F_i = \int_0^S ds \left\| \frac{d\hat{\boldsymbol{C}}(s)}{ds} \right\| f_i(s), \tag{33}$$

$$0 \;=\; \tau_i = \int_0^S ds \left\| \frac{d\hat{\boldsymbol{C}}(s)}{ds} \right\| \epsilon_{ijk}[C_j(s) - X_j^*] f_k(s), \tag{34}$$

---

[1] For quasi-2D motions along the surface, the contact angle $\theta_\epsilon$ remains constant and, hence, $\dot{\mathcal{R}}_x = 0$.

with $\boldsymbol{X}^*$ denoting the center of rotation, yields a $6\times6$-linear system which could be solved to obtain exact RFT- results for $\boldsymbol{U}$ and $\dot{\boldsymbol{N}}$. However, the resulting expressions are very complicated and do not offer much insight. Fortunately, it is possible to obtain simple analytical formulas for $\boldsymbol{U}$ and $\dot{\boldsymbol{N}}$, that capture the essential parts of their dynamics, by focussing on the two limit cases $\boldsymbol{U} \gg \dot{\mathfrak{R}}_{\boldsymbol{N}} \cdot \hat{\boldsymbol{C}}$ (translation-dominated regime) and $\boldsymbol{U} \ll \dot{\mathfrak{R}}_{\boldsymbol{N}}\hat{\boldsymbol{C}}$ (rotation-dominated regime).

To estimate $\boldsymbol{U}$, note that steric interactions between flagellum and channel wall compensate drag forces in vertical directions, so that only the $(x,y)$-components of the velocity are non-zero. Considering the translation-dominated regime $\boldsymbol{U} \gg \dot{\mathfrak{R}}_{\boldsymbol{N}} \cdot \hat{\boldsymbol{C}}$, the zero-force conditions (34) in the $(x,y)$-directions, $F_1 = 0$ and $F_2 = 0$, can be solved for $\boldsymbol{U} = (U_x, U_y)$. After averaging over $\phi$ with a uniform angular distribution, we find for $\epsilon \ll 1$ and $\kappa \simeq 1$ to leading order[2]

$$\boldsymbol{U} \simeq \frac{1}{2}\epsilon\,\sigma\dot{\gamma}\lambda S \begin{pmatrix}0\\1\end{pmatrix} - \frac{\chi}{3}\epsilon^2\,(\kappa-1)\,\sigma\dot{\gamma}\lambda S^2 \begin{pmatrix}0\\N_xN_y\end{pmatrix}, \tag{35}$$

where $\Lambda \simeq S\lambda$ is the approximate length of the flagellum. The first term is the mean drag on the geometric center of the conical helix, and the second is an orientation-dependent drag contribution due to chirality $\chi$. For passive chiral objects, such as dead bacterial cells, both terms can be important, although the first term is likely more relevant for self-swimming sperm cells. For completeness, we mention that the leading-order transverse-drag term (not shown) appears at next order in $(\kappa-1)$ and is found to be proportional to $-\chi\sigma(\kappa-1)^2\epsilon^2S^2\dot{\gamma}\lambda$.

Guided by Eq. (35), we simulate the position dynamics of sperm cells that swim at self-swimming speed $V$ in the direction of their 2D orientation $\boldsymbol{N}$ by implementing a minimal dynamics of the form

$$\dot{\boldsymbol{R}} = V\boldsymbol{N} + \boldsymbol{U} = V\boldsymbol{N} + \sigma\dot{\gamma}\epsilon\eta\begin{pmatrix}0\\1\end{pmatrix}, \tag{36}$$

where $\eta > 0$ is a geometric prefactor with dimensions of length. Neglecting the translational chirality-effects in Eq. (36) is indeed a reasonable approximation since, for sufficiently fast sperm cells, the beat chirality acts predominantly through the rotation dynamics of $\boldsymbol{N}$, which becomes amplified by multiplication with $V$ in Eq. (36).

To obtain an equation of motion for $\dot{\boldsymbol{N}}$, we first remark that due to conservation of $|\boldsymbol{N}|^2 = 1$, the dynamics of the components $\dot{N}_x$ and $\dot{N}_y$ are coupled by

$$0 = |\dot{\boldsymbol{N}}|^2 = 2(N_x\dot{N}_x + N_y\dot{N}_y). \tag{37}$$

Thus, only one of the three zero-torque conditions (34) is needed to determine both $\dot{N}_x$ and $\dot{N}_y$. For sperm swimming next to a solid surface, only rotations parallel to the surface are possible and, therefore, the relevant condition is $\tau_3 = 0$. Whilst a passive helix would rotate around its center of mass, the rotation axis is shifted towards the head position $\boldsymbol{R}$ for real sperm cells due to the presence of the cell head, which has been omitted thus far in our discussion of the rigid-spiral model. To account at least partially for the influence of the head on the rotation dynamics, we approximate $\boldsymbol{X}^* \simeq (\boldsymbol{R}, 0)$ in Eq. (34) and focus on the rotation dominated regime, $\boldsymbol{U} \ll \dot{\mathfrak{R}}_{\boldsymbol{N}} \cdot \hat{\boldsymbol{C}}$. Adopting these simplifications and averaging over $\phi$, one finds for small $\epsilon \ll 1$ from the vanishing $\tau_3$-component of Eq. (34) the leading order result

$$\dot{\psi} = \epsilon\,\dot{\gamma}\sigma\sin\psi + \frac{\chi}{4}\epsilon^2\,\frac{\kappa-1}{\kappa}\,\dot{\gamma}\sigma\,S\cos\psi. \tag{38}$$

Recalling that $\boldsymbol{N} = (N_x, N_y) = (-\sin\psi, \cos\psi)$, this can be rewritten as

$$\dot{\boldsymbol{N}} = \sigma\dot{\gamma}\epsilon\begin{pmatrix}N_xN_y\\N_y^2-1\end{pmatrix} + \frac{\chi}{4}\epsilon^2\,\frac{\kappa-1}{\kappa}\,\dot{\gamma}\sigma\,S\begin{pmatrix}N_x^2-1\\N_xN_y\end{pmatrix}. \tag{39}$$

The first term represents alignment against the flow due to the conical shape of the flagellar envelope, in agreement with Eq. (14). The second term describes chirality-induced deviations from exact anti-alignment, leading to a non-vanishing transversal velocity component, as observed in the experiments.

---

[2] The first term in Eq. (35) could also have been obtained by simply computing the mean drag velocity

$$\overline{\boldsymbol{u}} = \frac{1}{2\pi}\int_0^{2\pi} d\phi\left[\frac{1}{\Lambda}\int_0^S ds\,\left|\left|\frac{d\hat{\boldsymbol{C}}(s)}{ds}\right|\right|\,\boldsymbol{u}(\boldsymbol{C}(s))\right].$$

Clearly, the model of a rigid conical helix, as discussed here, is a relatively crude approximation to the full swimming dynamics of a sperm cell, for it neglects dynamical aspects of the flagellar beat (exact wave form, etc.) as well as hydrodynamic effects due to translation and rotation of the cell's head. Notwithstanding, it is plausible to expect that, on time scales larger than the typical beat period, Eqs. (36) and (39) provide a useful coarse-grained description of sperm swimming near a surface, as the model captures the main symmetries of the problem.

### Minimal model

We now summarise the minimal quasi-2D model implemented in our simulations. Assuming as before that the shear flow is along the $y$-axis (Fig. 1B of the Main Text), Eqs. (36) and (39) imply the following minimal 2D model for the quasi-2D motion of a sperm with position $\boldsymbol{R}(t) = (X(t), Y(t))$ and orientation $\boldsymbol{N}(t) = (N_x(t), N_y(t))$ in the vicinity of the surface

$$\dot{\boldsymbol{R}} = V\boldsymbol{N} + \sigma\overline{U}\boldsymbol{e}_y, \tag{40}$$

$$\dot{\boldsymbol{N}} = \sigma\dot{\gamma}\alpha \begin{pmatrix} N_x N_y \\ N_y^2 - 1 \end{pmatrix} + \sigma\dot{\gamma}\chi\beta \begin{pmatrix} N_x^2 - 1 \\ N_x N_y \end{pmatrix} + (2D)^{1/2}(\boldsymbol{I} - \boldsymbol{N}\boldsymbol{N}) \cdot \boldsymbol{\xi}(t). \tag{41}$$

Here, $V > 0$ is the self-swimming speed, $\sigma = \pm 1$ defines the flow direction, $\dot{\gamma} > 0$ is the shear rate, $\overline{U} > 0$ the mean flow speed experienced by the cell, and $\chi \in \{0, \pm 1\}$ the beat chirality. The dimensionless geometry parameters $\alpha > 0, \beta > 0$ encode details of the shape of the flagellar beat, and the coefficient $D$ determines the strength of the two-dimensional Gaussian white noise $\boldsymbol{\xi}$, interpreted here in the Stratonovich-sense and included to account for variability in sperm swimming.

For $D = 0$, the fixed points of Eq. (41) read

$$\boldsymbol{N}^+ = \left( \frac{\chi\beta}{\sqrt{\alpha^2 + \beta^2\chi^2}}, \frac{\alpha}{\sqrt{\alpha^2 + \beta^2\chi^2}} \right), \qquad \boldsymbol{N}^- = -\boldsymbol{N}^+. \tag{42}$$

The Jacobian

$$\mathcal{J}(\boldsymbol{N}) = \dot{\gamma}\sigma \begin{pmatrix} \alpha N_y + 2\chi\beta N_x & \alpha N_x \\ \beta N_y \chi & 2\alpha N_y + \chi\beta N_x \end{pmatrix} \tag{43}$$

has eigenvalues

$$\lambda(\boldsymbol{N}^\pm) \in \left\{ \pm\dot{\gamma}\sigma\sqrt{\alpha^2 + \beta^2\chi^2}, \pm 2\dot{\gamma}\sigma\sqrt{\alpha^2 + \beta^2\chi^2} \right\}. \tag{44}$$

This means that, for flow in negative $y$-direction corresponding to $\sigma = -1$, only $\boldsymbol{N}^+$ with $N_y^+ > 0$ is stable, whereas for flow in positive $y$-direction with $\sigma = +1$ only $\boldsymbol{N}^-$ with $N_y^- < 0$ is stable. Thus, in both cases, upstream swimming is stable, but for $\chi = \pm 1$ there also exists a transverse component which gives rise to the experimentally observed spiraling motion when the surface is closed to a cylinder (Fig. 1A of the Main Text).

### Parameter estimates

To obtain rough estimates for the parameters in Eq. (41), we relate the properties of sperm to those of the rigid helix model. Assuming that a typical beat contains roughly one or two full wavelengths, $S$ should lie in the range $[2\pi, 4\pi]$. For simplicity, we fix $S \sim 4\pi$ although $S$ is a complicated function of viscosity, temperature and so on. Then, for a typical flagellum of length $\ell \sim 60\mu$m, we may identify $\ell \sim \Lambda \simeq \lambda S$, which gives $\lambda \sim 5\,\mu$m.

To estimate the parameters $V$ and $\overline{U}$ on the rhs. of Eq. (40), we list in Table I the mean swimming speeds $v_{0\mu}$ at zero shear, $\dot{\gamma} = 0$, as measured in experiments for different viscosity values $\mu$; in simulations, we identify $V \sim v_{0\mu}$. A comparison of Eq. (40) with Eq. (35) further suggests that

$$\overline{U} \sim \frac{\dot{\gamma}}{2}\epsilon\Lambda \simeq \frac{\dot{\gamma}}{2}\epsilon\ell = \frac{\dot{\gamma}}{2}A, \tag{45}$$

where $A$ is the beat amplitude at the tail's end, corresponding to $||\hat{\boldsymbol{C}}(S)||$ in our model (i.e., the maximal separation of points visited by the head is roughly $2A$). Generally, this amplitude depends on viscosity, temperature and other parameters.

In the experiments, we measured mean sperm velocities $\langle \dot{\boldsymbol{R}} \rangle_{\dot{\gamma},\mu}$ at non-zero shear rate $\dot{\gamma}$ and different viscosities $\mu$. In the minimal model, the velocity components $\langle \dot{\boldsymbol{R}} \rangle = (\langle \dot{R}_x \rangle, \langle \dot{R}_y \rangle)$ are determined by

$$\langle \dot{\boldsymbol{R}} \rangle = V \langle \boldsymbol{N} \rangle + \sigma \overline{U} \boldsymbol{e}_y. \tag{46}$$

To obtain coarse estimates of the shape parameters $(\alpha, \beta)$ in Eq. (41), let us assume that for sufficiently weak noise one can approximate the mean orientation $\langle \boldsymbol{N} \rangle$ by the stable fixpoint of the deterministic equation[3] with $D = 0$, that is $\langle \boldsymbol{N} \rangle \simeq -\sigma \boldsymbol{N}^+$. Inserting this approximation into (46) and focussing on flow in negative $y$-direction (Fig. 1A of the Main Text), corresponding to $\sigma = -1$, we obtain

$$\begin{pmatrix} \langle \dot{R}_x \rangle \\ \langle \dot{R}_y \rangle \end{pmatrix} = \frac{V}{\sqrt{\alpha^2 + \beta^2 \chi^2}} \begin{pmatrix} \chi\beta \\ \alpha \end{pmatrix} - \begin{pmatrix} 0 \\ \overline{U} \end{pmatrix}. \tag{47}$$

For non-chiral quasi-planar beats with $\chi = 0$, this simplifies to the trivial relation

$$\begin{pmatrix} \langle \dot{R}_x \rangle \\ \langle \dot{R}_y \rangle \end{pmatrix} = \begin{pmatrix} 0 \\ V - \overline{U} \end{pmatrix}, \tag{48}$$

indicating that the transverse velocity vanishes. This scenario is roughly realised by bull sperm at very high viscosities (see Fig. 1C in the Main Text).

For chiral beats with $\chi^2 = 1$, we have

$$\begin{pmatrix} \langle \dot{R}_x \rangle \\ \langle \dot{R}_y \rangle \end{pmatrix} = \frac{V}{\sqrt{\alpha^2 + \beta^2}} \begin{pmatrix} \chi\beta \\ \alpha \end{pmatrix} - \begin{pmatrix} 0 \\ \overline{U} \end{pmatrix}. \tag{49}$$

In our experiments at low viscosities, we observe $\langle \dot{R}_x \rangle > 0$ for flow in negative $y$-direction with $\sigma = -1$, suggesting that we must choose $\chi = +1$ in our model to obtain qualitative agreement with the experimental data for bull sperm as well as for human sperm at low-to-moderate viscosity values, whereas for high viscosity values the handedness of human sperm changes to $\chi = -1$. To estimate $(\alpha, \beta)$, note that a comparison of Eqs. (39) and (41) suggests that $\alpha \approx \epsilon$ and which, when inserted into (45), gives

$$\overline{U} \simeq \frac{\dot{\gamma}}{2} \alpha \ell = u_{20} \frac{\alpha \ell}{2 \cdot 20 \mu\mathrm{m}} v_{0\mu}. \tag{50}$$

Assuming a flagellum length $\ell = 60\mu$m, identifying $V \approx v_{0\mu}$ with values $v_{0\mu}$ as given in Table I, and defining

$$v_{\mathrm{trans}} = \frac{\langle \dot{R}_x \rangle}{v_{0\mu}}, \qquad v_{\mathrm{up}} = \frac{\langle \dot{R}_y \rangle}{v_{0\mu}} \tag{51}$$

we can rewrite Eq. (49) as

$$\begin{pmatrix} v_{\mathrm{trans}} \\ v_{\mathrm{up}} \end{pmatrix} = \frac{1}{\sqrt{\alpha^2 + \beta^2}} \begin{pmatrix} \chi\beta \\ \alpha \end{pmatrix} - \frac{3u_{20}}{2} \begin{pmatrix} 0 \\ \alpha \end{pmatrix} \tag{52}$$

Data values for $(u_{20}, v_{\mathrm{trans}}, v_{\mathrm{up}})$ are given in Fig. 1D of the Main Text, yielding estimates for $(\alpha, \beta)$ as summarised in Tables II and III.

| Viscosity $\mu$ (mPa·s) | 1 | 3 | 12 | 20 |
|---|---|---|---|---|
| Human $v_{0\mu}$ ($\mu$m/s) | $53.5 \pm 3.0$ | $46.8 \pm 3.7$ | $36.8 \pm 3.3$ | $29.7 \pm 3.9$ |
| Bull $v_{0\mu}$ ($\mu$m/s) | $70.4 \pm 11.8$ | $45.6 \pm 4.7$ | $32.4 \pm 4.8$ | $29.6 \pm 4.1$ |

TABLE I: Sample mean speed $v_{0\mu}$ at zero shear $\dot{\gamma} = 0$ for different viscosities, averaged over different experiments.

[3] This assumption is, strictly speaking, only valid near the maxima of the curves in Fig. 1D of the Main Text.

| Viscosity $\mu$ (mPa·s) | 1 | 3 | 12 | 20 |
|---|---|---|---|---|
| Shear velocity $u_{20}$ | 1.0 | 1.1 | 1.4 | 1.2 |
| Transverse velocity $v_{\text{trans}}$ | 0.20 | 0.32 | -0.10 | -0.03 |
| Upstream velocity $v_{\text{up}}$ | 0.41 | 0.32 | 0.36 | 0.22 |
| Human $(\chi, \alpha, \beta)$ | (+1, 0.37, 0.08) | (+1, 0.38, 0.12) | (-1, 0.30 , 0.03) | (-1, 0.43, 0.01) |

TABLE II: Estimates of the shape parameters $(\alpha, \beta)$ in Eq. (41) for human sperm, as obtained from Eq. (52) for selected data values $(u_{20}, v_{\text{trans}}, v_{\text{up}})$ chosen near the maxima of the upstream velocity in Fig. 1D of the Main Text.

| Viscosity $\mu$ (mPa·s) | 1 | 3 | 12 | 20 |
|---|---|---|---|---|
| Shear velocity $u_{20}$ | 1.0 | 1.2 | 1.3 | 0.8 |
| Transverse velocity $v_{\text{trans}}$ | 0.09 | 0.12 | 0.17 | 0.03 |
| Upstream velocity $v_{\text{up}}$ | 0.49 | 0.37 | 0.14 | 0.04 |
| Bull $(\chi, \alpha, \beta)$ | (+1, 0.34, 0.03) | (+1, 0.35, 0.04) | (+1, 0.43, 0.07) | (+1, 0.80, 0.02 ) |

TABLE III: Estimates of the shape parameters $(\alpha, \beta)$ in Eq. (41) for bull sperm, as obtained from Eq. (52) for selected data values $(u_{20}, v_{\text{trans}}, v_{\text{up}})$ chosen near the maxima of the upstream velocity in Fig. 1D of Main Text.

## Simulations

We simulated the minimal model numerically by implementing the equivalent Ito-form of Eqs. (40) and (41),

$$\dot{\boldsymbol{R}} = V\boldsymbol{N} + \sigma\overline{U}\boldsymbol{e}_y, \tag{53}$$

$$\dot{\boldsymbol{N}} = \sigma\dot{\gamma}\alpha \begin{pmatrix} N_x N_y \\ N_y^2 - 1 \end{pmatrix} + \sigma\dot{\gamma}\chi\beta \begin{pmatrix} N_x^2 - 1 \\ N_x N_y \end{pmatrix} - D\boldsymbol{N} + (2D)^{1/2}(\boldsymbol{I} - \boldsymbol{N}\boldsymbol{N}) \cdot \boldsymbol{\xi}(t). \tag{54}$$

with an Euler discretisation scheme (time-discretisation $dt = 0.00005$ s). Since the noise enters multiplicatively in Eq. (41), the addition $(-D\boldsymbol{N})$-term is required within the Ito-scheme to ensure that Eq. (41) conserves the (average) length of the orientation vector $\boldsymbol{N}$.

The parameter estimates in Tables II and III were used as initial values for parameter scans. Generally, we found that, in the natural viscosity regime $\mu \sim 3$mPa, values $\alpha \in [0.2, 0.4]$, $\beta \in [0.05, 0.1]$ and $D \in [0.2, 0.3]\,\text{rad}^2/\text{s}$ yield good quantitative agreement with the experimental data (see Fig. 3 in the Main Text). Averages shown in Fig. 3A of the Main Text were obtained by starting from random initial conditions and integrating Eqs. (40) and (41) up to $t = 50\,$s. The following sperm parameters were adopted in Fig. 3 of the Main Text: self-swimming speed $V \sim 50\,\mu$m (cf. Table I), flagella length $\ell = 60\,\mu$m, and $\overline{U}$ was fixed by Eq. (45). Between simulation runs, the shear rate $\dot{\gamma}$ was varied over the same range as in the experiments. In spite of its relative simplicity, the model is capable of reproducing the main features of the experimental data both qualitatively and quantitatively.

---

[1] Pedley, T.J. Kessler, J.O. Hydrodynamic phenomena in suspensions of swimming microorganisms. *Annu. Rev. Fluid Mech.* **24**, 313–358 (1992).
